# IM- LTS: An Integrated Model for Lung Tumor Segmentation using Neural Networks and IoMT

**DOI:** 10.1016/j.mex.2025.103201

**Published:** 2025-02-07

**Authors:** Jayapradha J, Su-Cheng Haw, Naveen Palanichamy, Kok-Why Ng, Senthil Kumar Thillaigovindhan

**Affiliations:** a Department of Computing Technologies, School of Computing, SRM Institute of Science and Technology, Kattankulathur, Tamil Nadu 603203, India; b Faculty of Computing and Informatics, Multimedia University, Jalan Multimedia, 63100 Cyberjaya, Malaysia

**Keywords:** Lung Tumor Segmentation, Neural Networks (NN), Internet of Medical Things (IoMT), Support Vector Machine, Classification, Integrated Model (IM- LTS) for Lung Tumor Segmentation]

## Abstract

In recent days, Internet of Medical Things (IoMT) and Deep Learning (DL) techniques are broadly used in medical data processing in decision-making. A lung tumour, one of the most dangerous medical diseases, requires early diagnosis with a higher precision rate. With that concern, this work aims to develop an Integrated Model (IM- LTS) for Lung Tumor Segmentation using Neural Networks (NN) and the Internet of Medical Things (IoMT). The model integrates two architectures, MobileNetV2 and U-NET, for classifying the input lung data. The input CT lung images are pre-processed using Z-score Normalization. The semantic features of lung images are extracted based on texture, intensity, and shape to provide information to the training network.

• In this work, the transfer learning technique is incorporated, and the pre-trained NN was used as an encoder for the U-NET model for segmentation. Furthermore, Support Vector Machine is used here to classify input lung data as benign and malignant.
• The results are measured based on the metrics such as, specificity, sensitivity, precision, accuracy and F-Score, using the data from benchmark datasets. Compared to the existing lung tumor segmentation and classification models, the proposed model provides better results and evidence for earlier disease diagnosis.

## Graphical abstract

**Figure:**
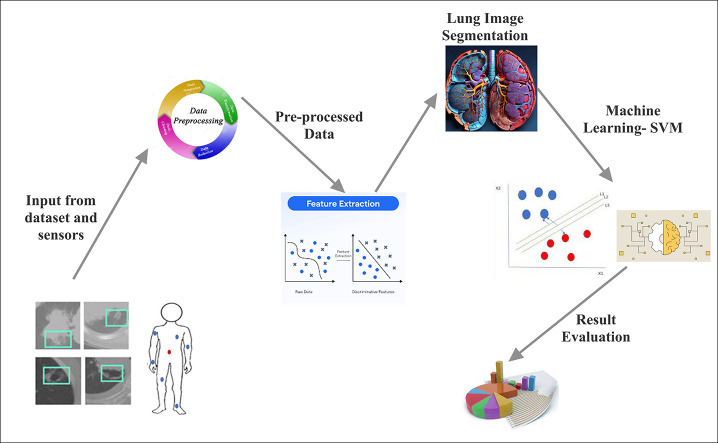

Specifications table

**Table:** 

Subject area:	Computer Science
More specific subject area:	Neural Network and IoMT
Name of your method:	Integrated Model (IM- LTS) for Lung Tumor Segmentation]
Name and reference of original method:	Transfer learning technique, NN, U-NET]
Resource availability:	Singh, S., Pais, A.R. & Crasta, L.J. Transfer Learning-Hierarchical Segmentation on COVID CT Scans. New Generation Computing, 42, 551–577 (2024). https://doi.org/10.1007/s00354–024–00240-x]

## Background

Lung cancer is the most destructive among other types of cancers that affect both genders [1]. Specifically, appropriate tumor segmentation at risk is vital, and if there are errors, it may lead to incorrect medical decisions. Hence, accurate cancer diagnosis and segmentation can effectively reduce the time required for treatment planning and rescheduling upon the tumor varies [2]. The convergence of the Internet of Things (IoT) [3], the Internet of Medical Things (IoMT) [4] and Artificial Intelligence (AI) [5] in the medical field provides an extensive range of preferences [6]. This work is concentrated on deep learning and IoMT-based disease classification and segmentation models for smart healthcare models. Because of the heterogeneity of tumor features, including size, appearance and shape, tumor classification and segmentation remain a big confrontation. The automatic segmentation of lung tumors from CT scan images may help physicians diagnose patients earlier and track the course of their illnesses more closely. Traditional automated tumor segmentation approaches primarily use feature engineering, which further extracts features from input images to train the classifier [7]. However, U-NET [8], a convolutional neural network, is considered one of the most sophisticated methods for precise pattern categorization of tumors, setting a new standard in biomedical image segmentation [9], due to its ability to identify pertinent information automatically. This paper introduces the Integrated Model for Lung Tumor Segmentation (IM-LTS), a segmentation model that combines the MobileNetV2 and U-NET. Here, the input lung images are pre-processed with the normalization technique, following the feature extraction process is carried out. Further, the SVM model is used to classify the processed inputs into benign and malignant categories.

To examine a lung tumor's malignancy and the likelihood that it will develop into lung cancer, a detailed evaluation of the tumor is necessary. The authors [10] presented a SVM based on the 3D matrix pattern technique to prevent the loss of structural and local information. The model could not distinguish between benign and malignant tumors because the 3D volume of tumors used the whole area of interest (ROI) for analysis and provided it as an input image for the algorithm's training. To extract potential nodules from segmented lung parenchyma, [11] used the rapid marching approach as part of their lung parenchyma segmentation procedure. The study in [12] sought to overcome the non-medical character of ImageNet features by integrating unclassified medical images of the same condition to reduce the dependence on ImageNet. This work demonstrated the necessity for smart technologies to assist physicians in the early diagnosis of breast cancer. As a result, the image method performed well in comparison to other research in this area, and a modified Xception model was used to categorize histological images of breast cancer into four groups. On the other hand, the effort [13] sought to create an artificial intelligence-based method for automatically recognizing COVID-19 in chest CT scans. The study used transfer learning to produce image-level representations (ILRs) based on a deep CNN. To capture nearby connections between ILR vectors, they image a neighboring aware representation (NAR) Various other works have analyzed using CT images [14,15].

The authors devised a generative adversarial network [16] for free-form 3D tumor to encode the characteristics from the slices of the CT image. The GAN is composed of an improved convolutional feature dilated gated generator and a hybrid loss function. The dilated generator helps to identify and enlarge the tumor in the lung. An image volumetric segmentation network for 3D volumetric medical segmentation, was presented in [17] using a comprehensive 3D methodology based on V-net [18]. The authors [19] utilized the R2U-Net and RU-Net models to perform medical image segmentation on various benchmarks. Two phases comprise the framework using 2D CNN to help with the CT reading procedure in the lung tumor segmentation approach: (1) Using a Faster R-CNN model for nodule candidature detection, a deconvolution layer was included to expand the feature map. (2) False positive reduction, which is based on training a classifier to lessen the false positive generated by the first step, was achieved by merging the candidates after the model was trained on the slices of the CT scan [20].

A proposal has been made in [21] for an accurate segmenting lung nodule by a new approach called Wavelet U-Net++. The hybrid approach of U-Net++ and wavelet pooling to identify the low and high end information in the image to improve the segmentation accuracy. The authors [22] developed a two-stage method: the first stage uses an adaptive ROI algorithm to undertake patch-wise exploration along the axial axis to estimate a tumor's starting size. The second step involves further investigation in the coronal and sagittal planes to rule out the presence of a malignant tumor in the removed area. Three potential detectors were specifically created to identify malignant lesions made up the algorithm image by [23] to increase the sensitivity of lesion detection. ConvNets calculated and processed the nodule candidates by averaging the tumor's location and likelihood. To extract fine-grained spatio-temporal information, the proposed method included a 3D encoder block and a recurrent block of ConvLSTM layers. A 3D decoder block was then used to rebuild the volumetric segmentation mask. A pre-trained AlexNet model has been used uniquely to identify aberrant brain areas in MRI images [24]. The authors added batch normalization layers to an already-trained model and used an extreme learning machine to replace the final layers. In addition, a chaotic bat algorithm was used to optimize the extreme learning machine and improve its classification performance, resulting in state-of-the-art outcomes for the identification of aberrant brain regions. The authors provided a theoretical foundation for comprehending data augmentation strategies [25]. The Markov process is a general model of augmentation in which kernels arise on by itself. First-order feature averaging and second-order variance regularization components may be used to approximate data augmentations. They also examined the augmentation techniques that change the models' capacity for learning. However, via adversarial training and geometric and color modifications, data augmentation increases the amount of the training dataset. The authors proposed an architecture for volumetric segmentation [26], in which a network from [27] was expanded by substituting three-dimensional operations for all two-dimensional ones. In addition to using data augmentation strategies during training, the proposed network had been programmed from the beginning. The intricate three-dimensional structure of the Xenopus kidney was used to evaluate the network's function, and the findings were positive. The authors [28] explored the applicability of transfer learning in numerous areas, including image processing and interpretation, because it allows the new model to leverage the learned features and representations to profit from prior knowledge. They also demonstrated how often pre-trained models from the ImageNet dataset are used in classification applications for diabetic retinopathy, skin cancer, and breast cancer. In addition, the authors looked into the issues in the datasets related to breast cancer and melanoma in more detail and offered possible fixes.

Transfer learning's shortcomings in the medical field because of the source and target problems not matching were covered in different research [29]. The research image dual transfer learning (DTL), a cutting-edge strategy that focuses on the convergence of patterns across two domains, solves this problem. The image method used four pre-trained models trained on two datasets: images of breast and skin cancer. A modest number of classified images from the target job and enough unclassified images of the same illness were used to fine-tune the final layers of the models. The outcomes of the experiments showed that the image method enhanced the functionality of every model. Though lot of research work has been carried on the lung tumor segmentation, the existing models find challenges with heterogeneity of tumors such as size, shape and appearance that leads to challenges on segmentation accuracy. Also, there is a lack of IoMT based system for lung cancer diagnosis. These research problems are addressed in this work.

## Method details

Theoretical Framework and Proposed Prototype.

### Working procedure of Integrated Model (IM-LTS) for lung tumor segmentation

**Fig. 1:**
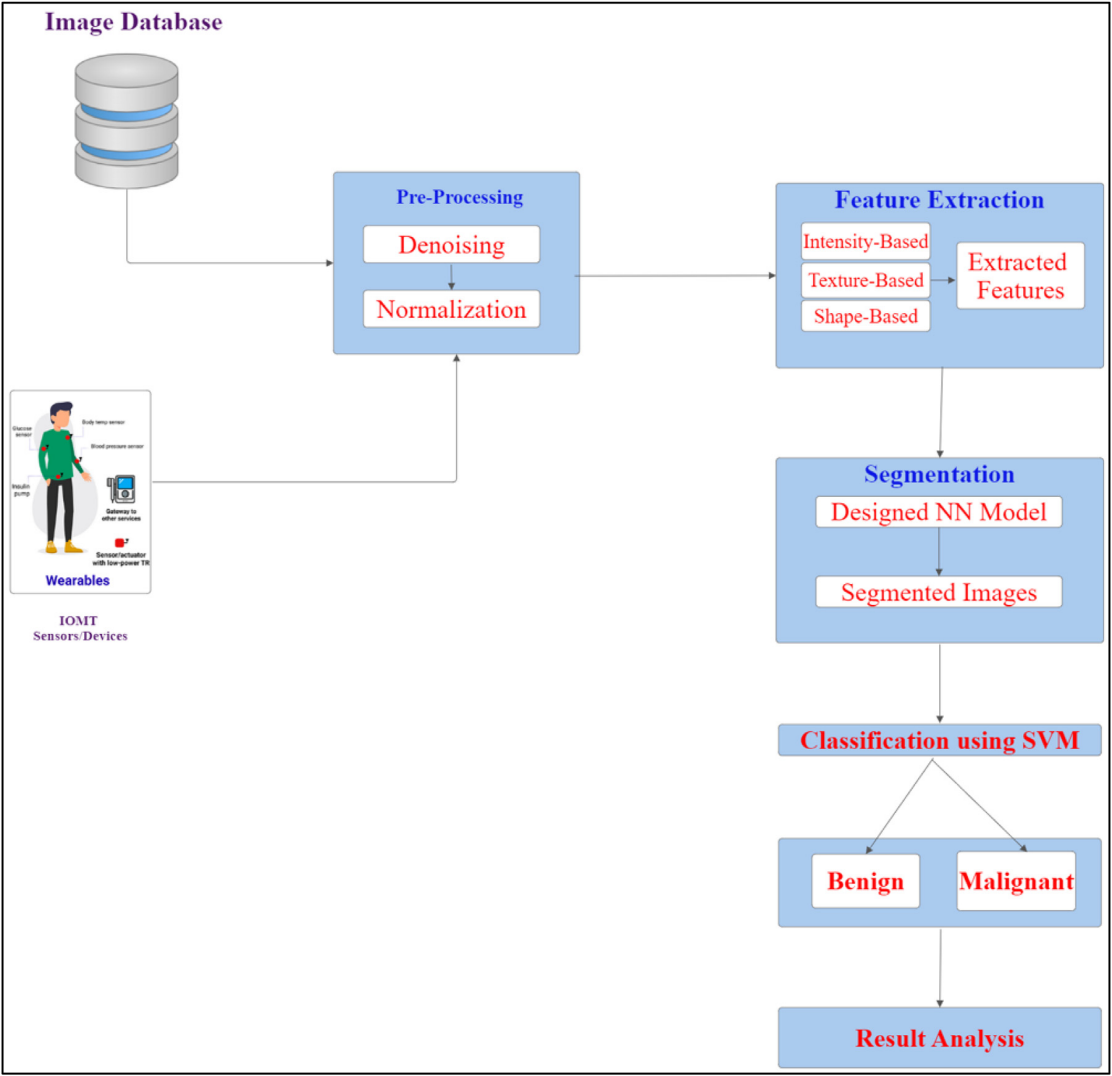
IM-LTS system architecture.

In this section, the working procedure of the proposed model is explained in detail. The proposed model is implemented using the inputs from IoMT based sensors and lung CT images. Here, the CT image data are obtained from Lung Image Database Consortium (LIDC), and the IoMT image data are obtained from the Linear Imaging and Self-Scanning Sensor (LISS) database. Fig. 1 displays the model of IM-LTS, in which the raw CT images are obtained from databases and pre-processed. Following, features are extracted and given to NN model for segmentation. Based on the size of the segmented tumor image, the classifications are made into two groups: benign and malignant.

In this section, the architecture is clearly defined by employing MobileNetV2 to solve the semantic segmentation problems. The proposed model is designed as a lightweight model with minimal features and attains an appropriate balance between the model outcome and implementation efficacy. The input image containing the lung nodule is provided as input for detecting the presence of tumor lesions using the proposed computations. The complete working process of IM-LTS is explained as follows.

### Pre-processing

Initially, the input images are pre-processed to enhance the images. The process obtains the input from IoMT sensors and database images. For pre-processing, the Bayesian threshold-based Taylor series approach [30] is used for de-noising. This technique provides high-resolution wavelet sub-bands to generate better quality pixels. The series is defined as the infinite addition of values that are computed from derivative functions, where the terms are pixels and diverse. For a 3D image, the Taylor series is given in Eq. (1).

(1) $$T\left(A\right)=\left(x\right)+{\left(A-x\right)}^{T}\text{Df}\left(x\right)+\frac{1}{2!}{\left(A-x\right)}^{T}\left\{D^{2}f\left(x\right)\right\}\left(A-x\right)+\ldots$$

The input consists of different rates of pixels as the image intensities are not equal at all places. Hence, the denoising process is very significant in processing lung images. Based on the definition of the Taylor series, the sub-band noise is determined and removed when the value is larger than the derived value of the Bayesian Threshold. The formula for threshold derivation ‘$T_{\text{Bay}}$’ is given as in Eq. (2).

(2) $$T_{\text{Bay}}=\frac{\sigma_{N}^{2}}{\sigma_{\text{sn}}}$$

Where, $\sigma_{N}$ denotes the noise derivation, and ‘$\sigma_{\text{sn}}$’ is the sub-band noise, and it is non-zero, can be given as in Eq. (3).

(3) $$\sigma_{\text{sn}}=\sqrt{\text{max}\left(\left(\sigma_{b}^{2}-\sigma_{b}^{2}\right),\;0\right)}$$

Where, $\sigma_{b}$ can be formulated as in Eq. (4)

(4) $$\sigma_{b}=\frac{1}{N}\left(\sum sn_{i}\right)$$

The sub-bands are given as in Eq. (5)

(5) $$sn_{i}=\left\{\text{LLL},\;\text{LLH},\;\text{LHL},\;\text{LHH},\;\text{HLL},\;\text{HLH},\;\text{HHL},\;\text{HHH}\right\}$$

From that, the total number of sub-bands ($\sigma_{N}$) is computed as in Eq. (6)

(6) $$\sigma_{N}=\frac{\text{median}\left(\text{sn}\right)}{0.6745}$$

According to the derived ‘$T_{\text{Bay}}$’ value, and the resultant values are sorted, and Eq. (7) can be framed using curve-fitting.

(7) $$\gamma =\frac{\varrho_{1}\delta^{2}+\varrho_{2}\delta^{1}+\varrho_{3}}{\delta +\rho }$$

In Eq. (7), ‘δ’ denotes the standard noise deviation, and ϱ and ρ are the constant factors. Further, filtering algorithm is applied, and the expression is given as in Eq. (8).

(8) $$\hat{B}S_{\text{sn}}=T_{3D}^{-1}\left(\propto \left(T_{3D}\left(X_{\text{sn}}\right),\;T_{\text{Bay}}\gamma \sqrt{2\text{log}\left(N^{2}\right)}\right)\right)$$

In Eq. (8), ‘$\hat{B}S_{\text{sn}}$’ the stacked sub-bands, $T_{3D}$ represents the unitary image transform, ‘X’ is the size (sub-bands), ‘∝’ threshold function. From this final equation, each sub-band is reframed, and the denoised images are attained. Next, the denoised images are given for feature extraction.

### Feature extraction

The features are extracted from the filtered lung images based on intensity, shape and texture.

### Intensity-based lung image features

This includes the features based on color intensity histograms and grayscale visibilities presented in the image. The features are correlation, contrast, homogeneity and entropy.

i. Correlation: It is measured based on similar directions of horizontal and vertical lines as depicted in Eq. (9).
  (9) $$\text{Imag}e_{\text{corr}}=\sum_{x,y=0}^{n-1}I_{x,y}{\left(x-y\right)}^{2}$$
ii. Contrast: It is measured based on the brightness of each lung nodule. The formula is given as in Eq. (10).
  (10) $$\text{Imag}e_{\text{con}}=\sum_{x,y=0}^{n-1}I_{x,y}\left(\frac{\left(x-\mu_{x}\right)\left(y-\mu_{y}\right)}{\sigma_{x}\sigma_{y}}\right)$$
iii. Homogeneity: It is computed based on the distribution proximity of matrix [31] entities. It is measured in all image directions as shown in Eq. (11).
  (11) $$\text{Imag}e_{\text{hom}}=\sum_{x,y}\frac{I\left(x,y\right)}{1+\left|x-y\right|}$$
iv. Entropy: It can be measured using the complexity of inequalities and image textures as depicted in Eq. (12).
  (12) $${\text{Image}}_{\text{ent}}=-\sum_{g=1}^{G}\sum_{g=1}^{G}I\;\left(g,g^{\prime }\right)\log I\;\left(g,g^{\prime }\right)$$

From the above equations, ‘I(x,y)’ is the normalized image matrix for a pixel and ‘n’ denotes the varied gray-levels in the image.

### Shape-based lung image features

This includes features based on the shape and size of tumor cells. The features are extracted from the processed image according to area, aspect rate, roundness, perimeter and circularity.

i. Area: The number of pixels present in the largest axial segment derives the area multiplied by the pixel resolution. The formula is given in Eq. (13).
  (13) $$\text{Imag}e_{\text{area}}=I\left(x,y\right)\;\times \;\Delta N_{a}$$
ii. Aspect Rate [32]: The aspect rate is measured with a major and minor axis length, and the formula is given in Eq. (14).
  (14) $$\text{Imag}e_{\text{AR}}=\frac{\text{MJ}\left(x,y\right)\left|L\right|}{\text{MN}\left(x,y\right)\left|L\right|}$$
iii. Roundness: This is the important feature measured based on the likeness of the lung nodule segment to the circular shape. It can be computed using Eq. (15).
  (15) $$\text{Imag}e_{R}=\frac{4\pi N_{a}}{L}$$
iv. Perimeter: Perimeter is the structural attribute for the coordinate sets and the sum of distance among coordinates. It can be derived for Eq. (16).
  (16) $$\text{Imag}e_{P}=\sqrt{{\left(M_{i}-M_{i-1}\right)}^{2}+{\left(N_{i}-N_{i-1}\right)}^{2}}$$
v. Circularity: It can be computed based on the circular area of each lung nodule. The computation is given in Eq. (17).
  (17) $$\text{Imag}e_{\text{cir}}=\frac{4\pi N_{a}}{D^{2}}$$

In the above set of equations, ‘$\Delta N_{a}$’ denotes the pixel area in I(x,y), $M_{i}$, $N_{i}$ is the ith pixel coordinates, ‘$N_{a}$’ nodule area, nodule segment boundary length is denoted by ‘L’, MJ(x,y)|L|, MN(x,y)|L| defines the length of the major axis and minor axis, respectively.

### Texture-based lung image features

The image intensity differences are considered as the texture here, comprising properties such as regularity, smoothness and unevenness. The texture-based features include uniformity, mean, standard variance, kurtosis, skewness and smoothness.

Uniformity: It can be measured using the uniform intensity of histograms, and the formula is given in Eq. (18).

(18) $$\text{Imag}e_{\text{Uni}}=\sum_{i=0}^{l-1}HS^{2}\left(P_{i}\right)$$

Mean: It can be defined as the average rate of intensity using Eq. (19).

(19) $$\text{Imag}e_{M}=\sum_{i=0}^{l-1}P_{i}\times \;\text{HS}\left(P_{i}\right)$$

Standard Variance: It can be measured from the second moment of the mean rate, using Eq. (20).

(20) $$\text{Imag}e_{\text{SV}}=\sum_{i=0}^{l-1}{\left(P_{i}-\text{moment}\right)}^{2}\times \;\text{HS}\left(P_{i}\right)$$

Kurtosis: It can be measured from the fourth moment of mean rate, using Eq. (21).

(21) $$\text{Imag}e_{K}=\sum_{i=0}^{l-1}{\left(P_{i}-\text{moment}\right)}^{4}\times \;\text{HS}\left(P_{i}\right)$$

Skewness: It can be computed from the third moment of mean rate using Eq. (22).

(22) $$\text{Imag}e_{\text{SK}}=\sum_{i=0}^{l-1}{\left(P_{i}-\text{moment}\right)}^{3}\times \;\text{HS}\left(P_{i}\right)$$

Smoothness: It defines the relative intensity variations in the provided segment based on Eq. (23).

(23) $$\text{Imag}e_{\text{SM}}=l-\frac{1}{1+\sigma^{2}}$$

In the above equations, ‘$P_{i}$’ is the random variable of intensity, ‘$\text{HS}(P_{i})$’ is the histogram of intensity, ‘l’ is the number of potential intensity rates and ‘σ’ is the standard deviation. The derived features are given to the network model for segmentation and classification.

### Network model

The network model comprises the traditional U-NET and MobileNetV2, in which the U-NET is the encoder-decoder-based architecture. Moreover, MobileNetV2 is the pre-trained encoder, and CNN is the U-NET model employed for medical image processing and analysis. Here, the pre-trained encoder uses lightweight convolutions to attain precise results with minimal features. Skip connections are also employed with the activation function called Relu [33] to maximize the network's convergence for connecting the encoder and decoder layers. The connections permit feature map concatenation with varied spatial resolutions. The input image is given to the encoder to derive the features, and the decoder is trained to produce the respective probability maps. Additionally, the down-sampling path skip connections are concatenated with the up-sampling feature maps for submitting the local data to global data.

**Fig. 2:**
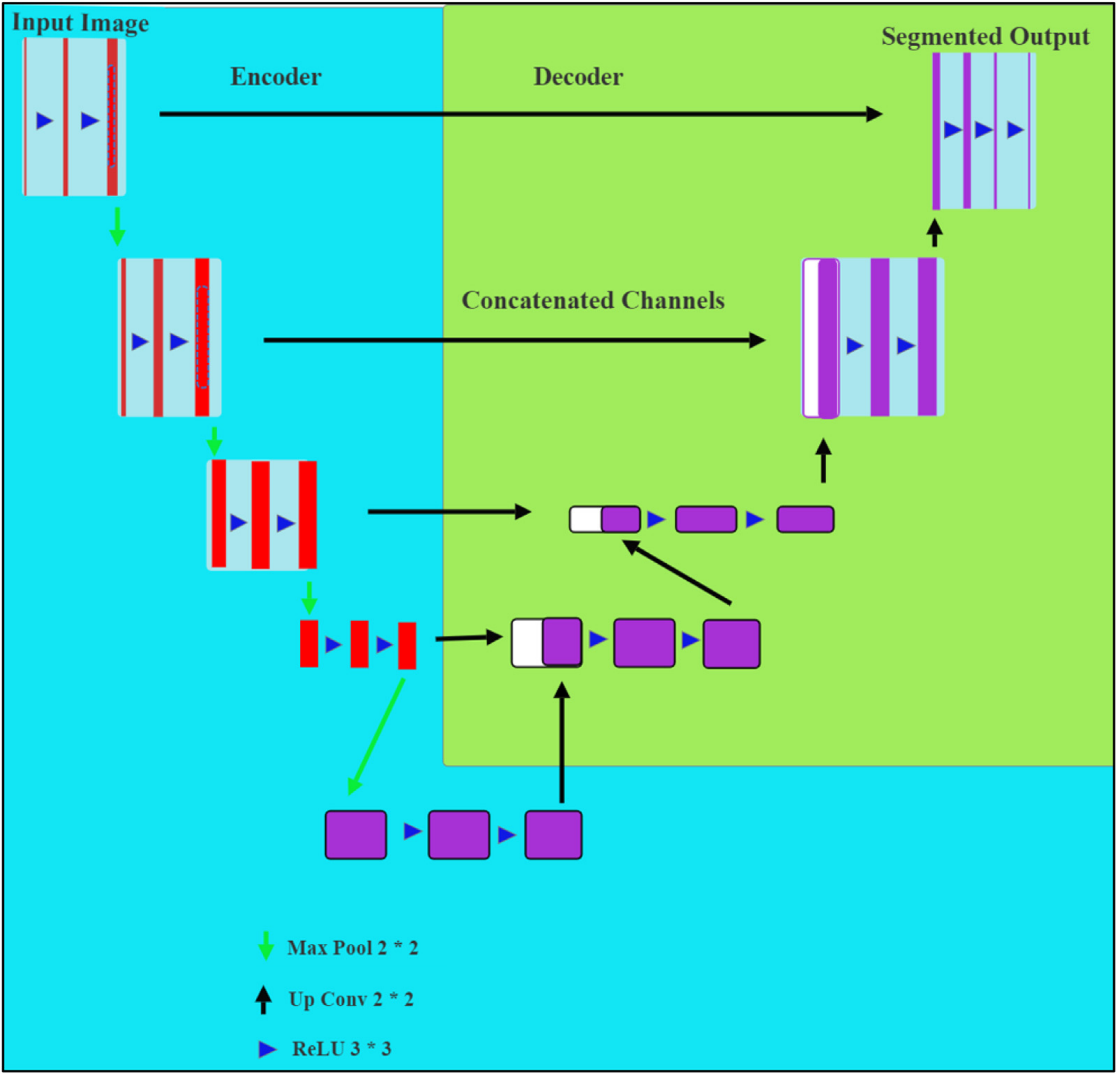
Network model (MobileNETV2 and CNN as backbone encoder).

While training the model, a total 90 epochs is processed with patch size 256×256, in eight batches. The fine-tuned hyperparameters are given in Table 1. The dice loss outperforms binary cross-entropy loss [34] and Jaccard loss [35] concerning the model performance and inclination for true positive rates. The high saturation of binary cross-entropy loss in input images has occurred by large patches of black pixels. The hybrid network model used the obtained generic image features as the pre-trained weights are initialized and trained with the large dataset images. When the validation loss performs a non-consistent drop in four epochs, the learning rate is reduced by 0.01. Moreover, after 10 epochs, the training process will be ended when the validation dice loss remains unchanged. Additionally, the obtained weights are updated using the Adam optimizer to enhance the model's learnability. A shortcut link was added to enable the gradient flow and to enhance feature reuse and model efficacy. The tumor nodule segmentation results are obtained using the network model and given as input to SVM for classifying the cancer stage. The network model is portrayed in Fig. 2.

**Table 1:** Hyper-parameters of designed network model.

Parameter	Value
Input Size	255×255
Batch Size	8
Learning Rate	0.0001
Optimizer	ADAM
Epoch	90
Activation Function	Sigmoid
Loss Function	LDice

### Lung tumor classification using SVM

Support Vector Machine (SVM), a supervised learning model, is used to classify the lung images after the nodule segmentation process. Additionally, the support vector machine is used in binary classification to estimate the Optimal Separating Hyperplane (OSH) [36], which creates maximum margin lanes among two classes of input images. A transformation is a non-linear mapping of a data element into a high-dimensional space. Additionally, it provides linear classification in the original image space.

An SVM creates a non-probabilistic binary linear classifier model by collecting and classifying input data components. Additionally, the classifier employs the Kernel function to map the input image into several spaces, allowing for efficient classification even in situations with intricate boundary conditions. Kernel functions, including radial basis, polynomial, and linear kernel functions, are used to build the classifier. In such cases, each kernel function is generated by its parameters. Assuming the training dataset $D=\{a_{i}-b_{i}\}$, from which each input relates to its respective high-dimensional feature space. Further, the radial basis function for tumor classification is given as in Eq. (24).

(24) $$R\left(a,b\right)=\text{exp}\left(-\frac{∥a-b∥}{2}s^{2}\right)$$

Where, ‘s’ is the positive real number. For classifying the inputs into benign and malignant levels, the segmented image, which is ≥3mm is classified as malignant, and those with <3mm are considered as benign. Cancer is categorized into classes based on a study of the medical sector to better aid in the treatment process.

### Dataset description

As mentioned above, the CT image data are obtained from the Lung Image Database Consortium (LIDC) [37], and the IoMT Image data are obtained from the Linear Imaging and Self-Scanning Sensor (LISS) [38] database, which are explained in the following sub-sections.

#### LISS dataset

This is a public database from which the images are used for research purposes. The dataset comprises 166 3D images, 511 2D CISLs, and approximately 9 lung nodule symptoms are observed from the data of the Cancer Institute and Hospital at Chinese Academy. The data were obtained from GE LightSpeed VCT-64 and Toshiba Aquilion 64 Slice CT Scanners. Moreover, the images in the database are stored in DICOM 3.0 format. Here, the 2D image samples are wrapped by 9 types of lung nodule mark images, and the 3D samples are covered by single nodule signs.

**Fig. 3:**
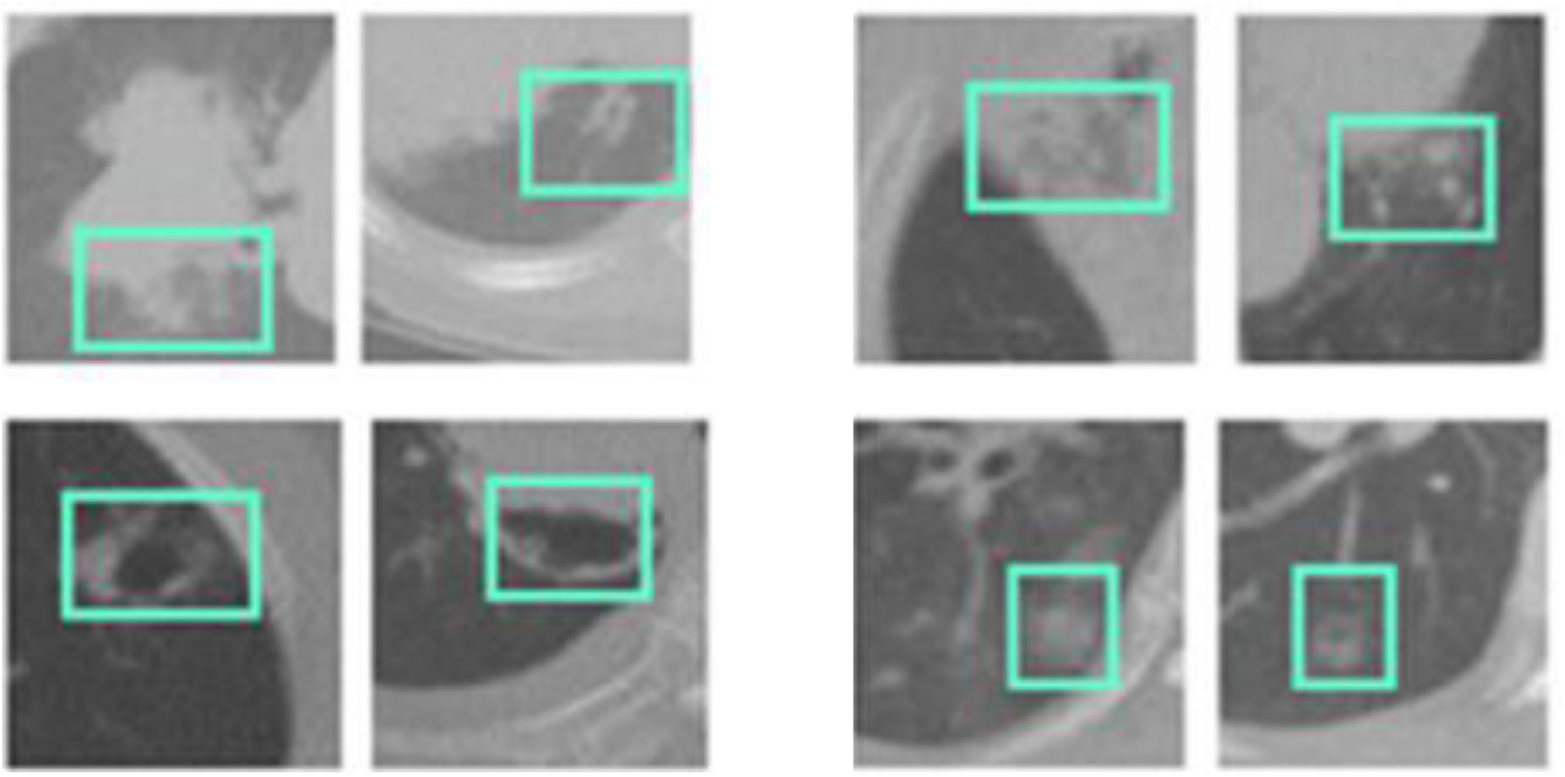
Sample images from LISS dataset (Abnormal signs are marked).

The CT images are provided with their respective labels, the image dimensions are measured in pixels, and the images in database are huge and have varied nodule signs. The slice size of each lung image is 0.418–1 mm, and the average size rate of each image is 0.664 mm. The description of the dataset is depicted in Table 2, comprising two sections: CT images and Annotated RoIs, while the sample images are shown in Fig. 3.

**Table 2:** LISS description.

Parameter	CT Images	Annotated RoIs
Ground Glass Opacity	2D- 25 3D-19	2D-45 3D-166
Lobulation	21	41
Cavity and Vacuoles	75	147
Pleural Dragging	26	80
Obstructive Pneumonia	16	18
Air Bronchogram	22	23
Speculation	18	29
Bronchial Mucus Plugs	19	81

#### LIDC-IDRI dataset

**Fig. 4:**
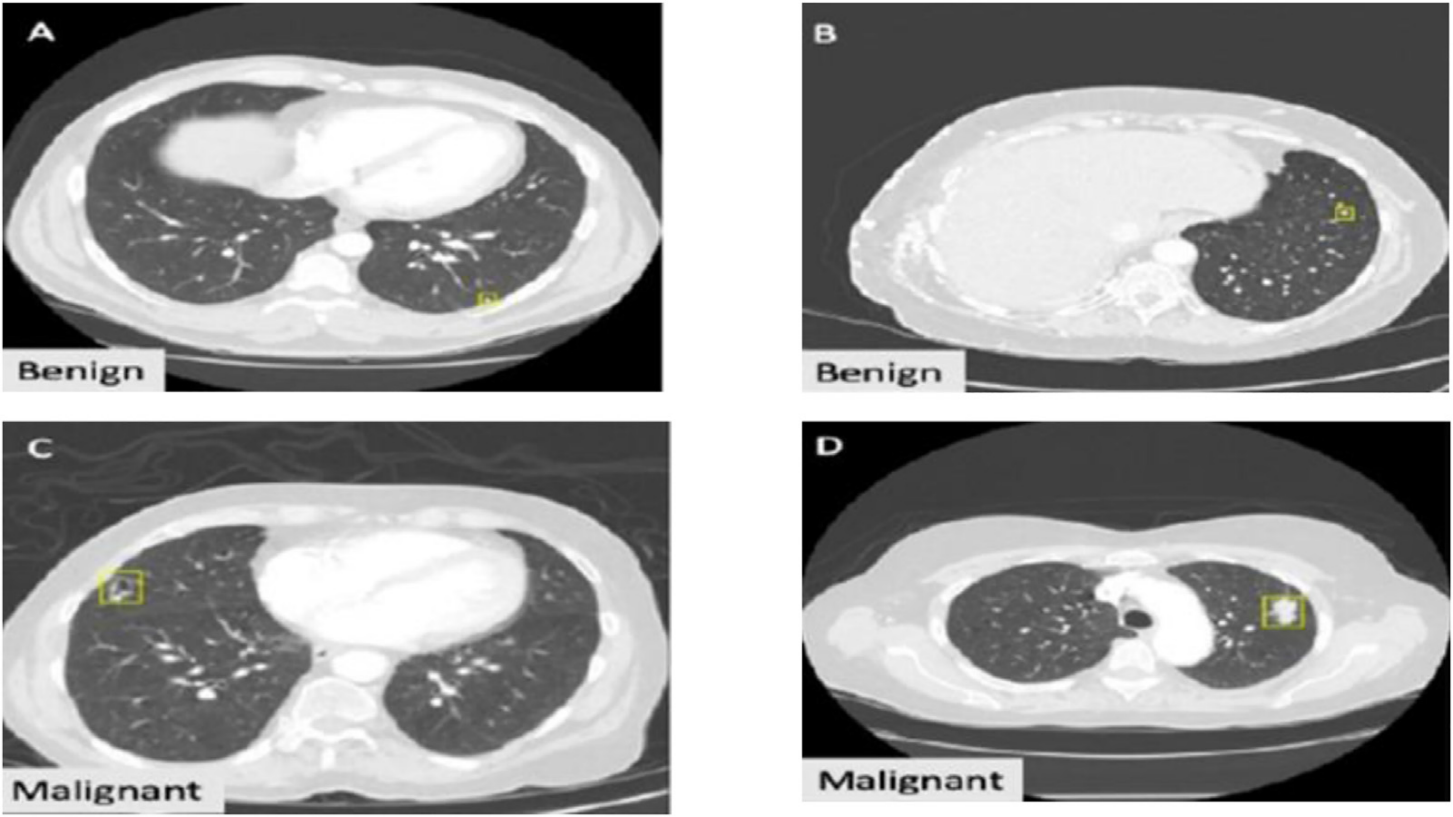
Sample images from LIDC-IDRI dataset.

This dataset is widely available and used by several researchers. It contains 1018 CT images from 1010 patients. The images demonstrate the occurrence of lung cancer and nodule annotations. Moreover, the ratings are assigned for each sample based on the disease progression, biopsy, surgical resection, and review of radiological observations. The descriptions of size-based image distribution are presented in Table 3. Several lung nodules are presented in varied lung images; each contains annotated data based on its size. Moreover, the database assigns size-based annotations for each nodule ranging from 3 mm to 30mm. The sample images are given in Fig. 4.

**Table 3:** LIDC-IDRI description.

Description	CT Images
No. Of nodules signed by ≤1 radiologist	7371
No. Of <3 mm nodules signed by ≤1 radiologist	2669
No. Of <3 mm nodules signed by =4 radiologist	928

### Method validation

The proposed IM- LTS model is analyzed and tested with the LIDC-IDRI lung CT dataset and LISS dataset comprising various CT scan images of patients with and without lung cancer and the images obtained from IoMT sensors, respectively. Furthermore, the implementation has been done with MATLAB V12 with an initial setting of 0.001 as its learning rate of classification. Moreover, the obtained results are compared with two existing models such as Faster R-CNN [39], random forest (RF) [40] and Dual Transfer Learning (DTL) [41] to prove the efficiency of the proposed work. The results are evaluated based on metrics such as, sensitivity (True Positive Rate), Specificity (Recall), Precision and Accuracy Rate, and their computations are given below.

#### Sensitivity

It is likelihood that the benign class will be correctly categorized in the same class as depicted in Eq. (25).

(25) $$\text{Sensitivity}=\left(\frac{\text{TP}}{\text{TP}+\text{FN}}\right)\times \;100\%$$

#### Specificity or recall

Its definition is the quantity of images classified as belonging to the malignant class; the derivation is shown in Eq. (26).

(26) $$\text{Specificity}=\left(\frac{\text{TP}}{\text{TN}+\text{FP}}\right)\times \;100\%$$

#### Precision rate (PR)

It is calculated using the categorization of lung CT scans into benign and malignant groups (see Eq. (27)).

(27) $$\text{PR}=\left(\frac{\text{TP}}{\text{TP}+\text{FP}}\right)\times \;100\%$$

#### Accuracy rate (AR)

The accuracy rate of image classification is used to assess the quality of the IM-LTS based on Eq. (28).

(28) $$\text{AR}=\left(\frac{\text{TP}+\text{TN}}{\text{TP}+\text{TN}+\text{FP}+\text{FN}}\right)\times \;100\%$$

**Fig. 5:**
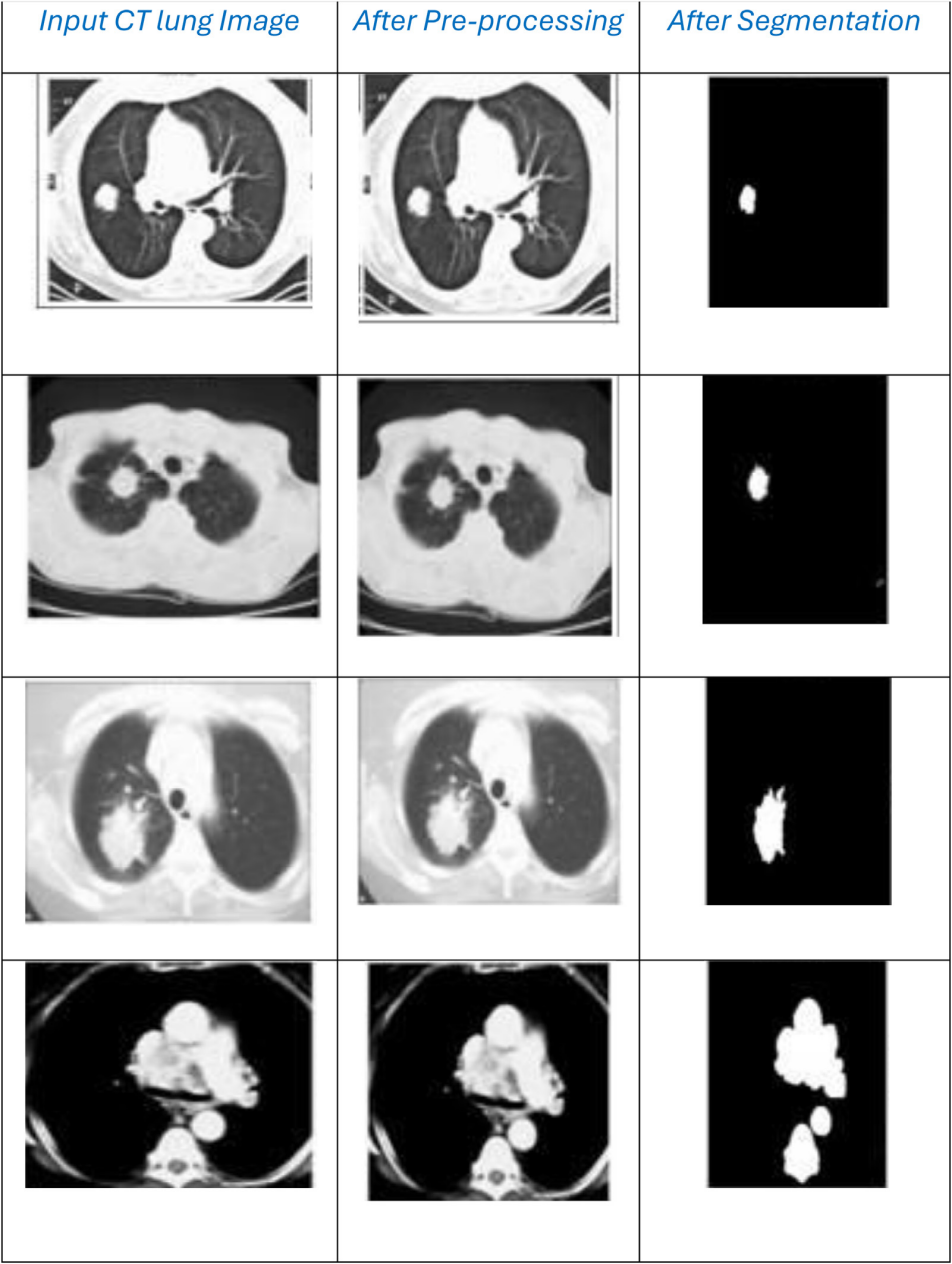
Results after pre-processing and segmentation.

Based on the Criteria discussed above, the results are clearly illustrated in Fig. 5.

Analysis criteria, including Sensitivity, Specificity, Precision Rate, Accuracy Rate, and Error Rate are used to accomplish the assessments. The findings of the sensitivity rate on 500 lung CT scans from the LISS and LIDC-IDRI datasets, respectively, are shown in Fig. 6 and Fig. 6a. The evaluations are processed with the varying rates of number of dataset images as {100, 200, 300, 400, 500}. The overall observations for model evaluations are presented in Table 4. The model attains results as, {85.1, 85.1,78.2, 89.8, 94.6} and {88.5, 93.2, 81.3, 91.5, 94.5}, for LIDC and LISS, respectively, for sensitivity. The image cancer diagnostic model has an average sensitivity rate of 94.693 %, higher than that of previous comparable research. The findings for the specificity rate of the image and current models are shown in Fig. 7 and Fig. 7a. The exactness of correct classification is successfully obtained in the image approach by effectively enforcing the deep learning model for tumor tissue segmentation and classification. Additionally, the model successfully classifies malignant images under that specific class with a greater rate of specificity, obtains, {97.1, 83.9, 88.9, 89.8, 90.2} and, {90.7, 88.1, 81.6, 88.1, 93.6} for LIDC and LISS, respectively, and its average sensitivity of 94.01 % is higher than other models, demonstrating its efficacy.

**Fig. 6:**
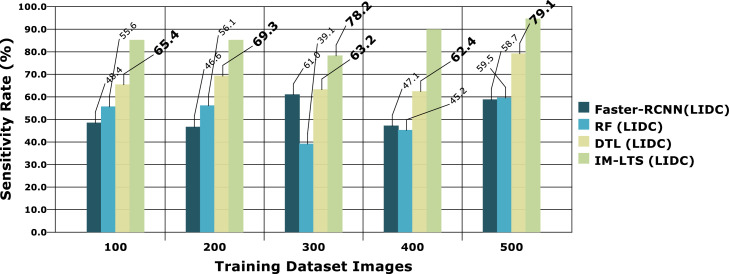
Sensitivity rate based evaluations for LIDC.

**Fig 6a:**
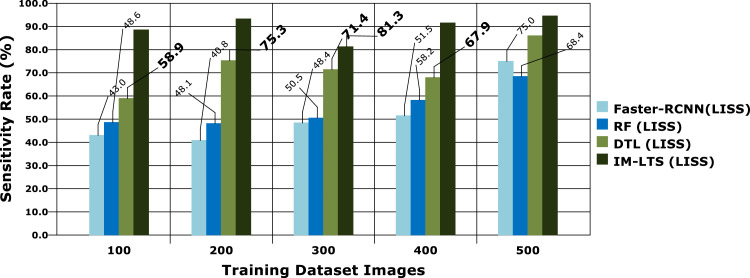
Sensitivity rate based evaluations for LISS.

**Table 4:** Comparison Results for LIDC and LISS Dataset Samples

Models	100	200	300	400	500
A. Sensitivity Rate					
Faster- RCNN (LIDC)	48.4	46.6	61.0	47.1	58.7
Faster- RCNN (LISS)	43.0	40.8	48.4	51.5	75.0
RF (LIDC)	55.6	56.1	39.1	45.2	59.5
RF(LISS)	48.6	48.1	50.5	58.2	68.4
DTL (LIDC)	65.4	69.3	63.2	62.4	79.1
DTL (LISS)	58.9	75.3	71.4	67.9	86.0
IM-LTS (LIDC)	85.1	85.1	78.2	89.8	94.6
IM-LTS (LISS)	88.5	93.2	81.3	91.5	94.5


B. Specificity Rate					
Faster- RCNN (LIDC)	55.4	64.6	48.5	64.7	52.4
Faster- RCNN (LISS)	52.5	73.4	57.9	80.3	58.6
RF (LIDC)	55.5	63.2	52.2	72.8	65.0
RF(LISS)	60.0	45.2	62.3	62.5	52.8
DTL (LIDC)	66.2	71.4	69.2	71.3	71.3
DTL (LISS)	71.0	58.9	76.0	76.9	75.1
IM-LTS (LIDC)	97.1	83.9	88.9	89.8	90.2
IM-LTS (LISS)	90.7	88.1	81.6	88.1	93.6


C. Rate of Accuracy					
Faster- RCNN (LIDC)	64.3	50.5	52.1	56.1	63.9
Faster- RCNN (LISS)	53.7	56.3	42.9	58.4	54.8
RF (LIDC)	60.8	48.7	57.6	52.6	73.7
RF(LISS)	64.0	61.1	65.5	58.2	56.8
DTL (LIDC)	68.4	69.6	85.1	74.8	82.1
DTL (LISS)	58.5	78.3	77.4	84.7	67.5
IM-LTS (LIDC)	89.0	93.7	92.4	92.0	93.7
IM-LTS (LISS)	95.4	96.3	96.7	95.4	95.8


D. Precision Rate					
Faster- RCNN (LIDC)	59.6	60.6	65.0	53.3	73.3
Faster- RCNN (LISS)	72.2	52.1	71.9	62.0	78.7
RF (LIDC)	57.7	66.8	67.4	69.7	79.2
RF(LISS)	64.6	73.7	75.5	74.7	66.4
DTL (LIDC)	70.1	79.9	75.6	81.5	57.1
DTL (LISS)	61.5	66.9	69.1	73.4	76.6
IM-LTS (LIDC)	81.5	88.6	87.5	85.8	91.2
IM-LTS (LISS)	90.6	96.6	93.4	95.0	98.3


E. Error Rate					
Faster- RCNN (LIDC)	34.8	37.3	34.5	44.6	40.3
Faster- RCNN (LISS)	44.1	43.9	42.2	46.2	48.4
RF (LIDC)	38.2	50.5	60.0	39.4	35.3
RF(LISS)	20.8	27.8	44.7	29.2	38.8
DTL (LIDC)	45.2	45.7	32.8	49.6	51.7
DTL (LISS)	60.9	57.7	27.3	31.1	34.4
IM-LTS (LIDC)	13.9	10.5	14.3	8.4	9.5
IM-LTS (LISS)	6.3	13.8	13.3	10.0	7.9

**Fig. 7:**
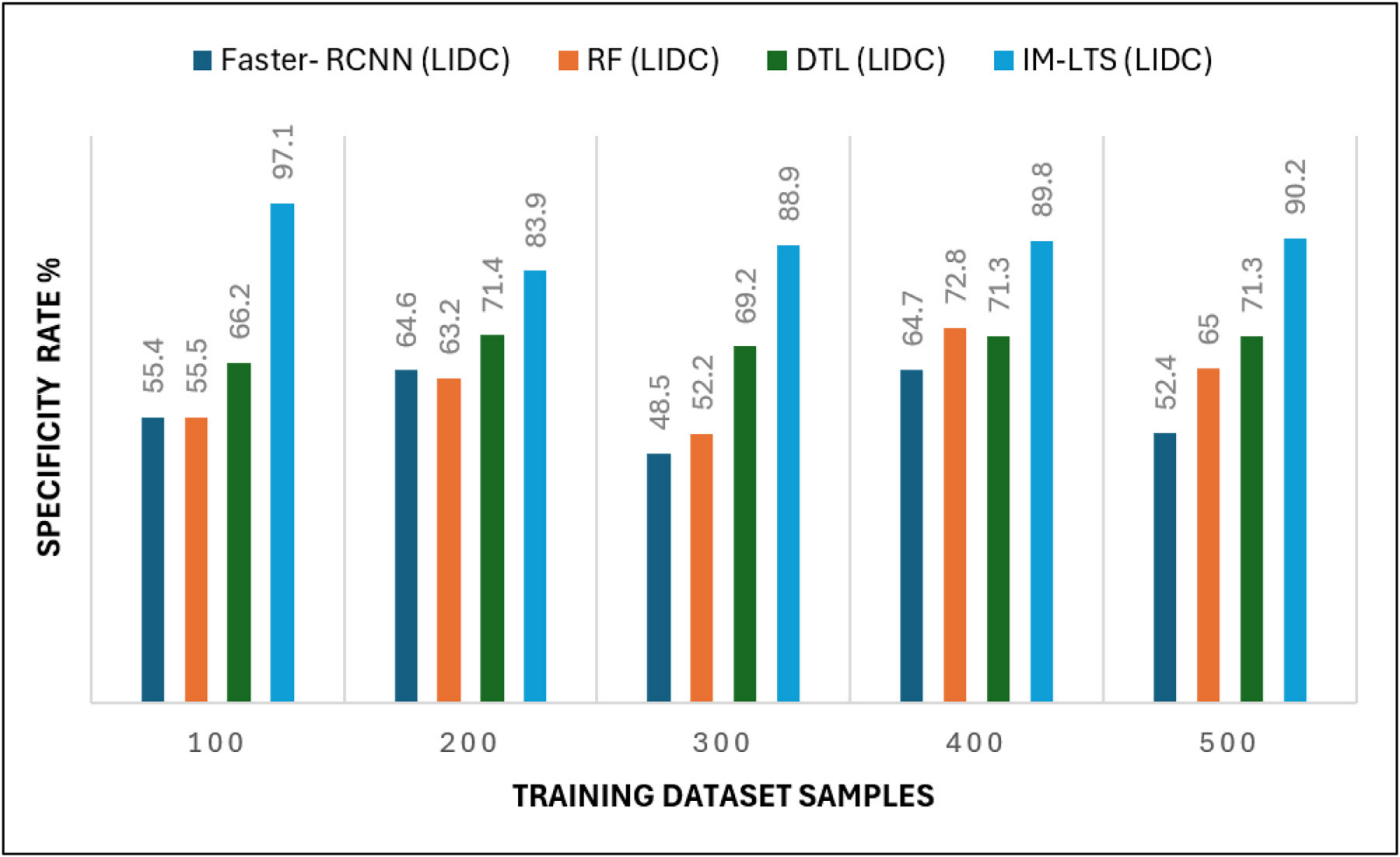
Specificity rate based comparisons and results for LIDC.

**Fig 7a:**
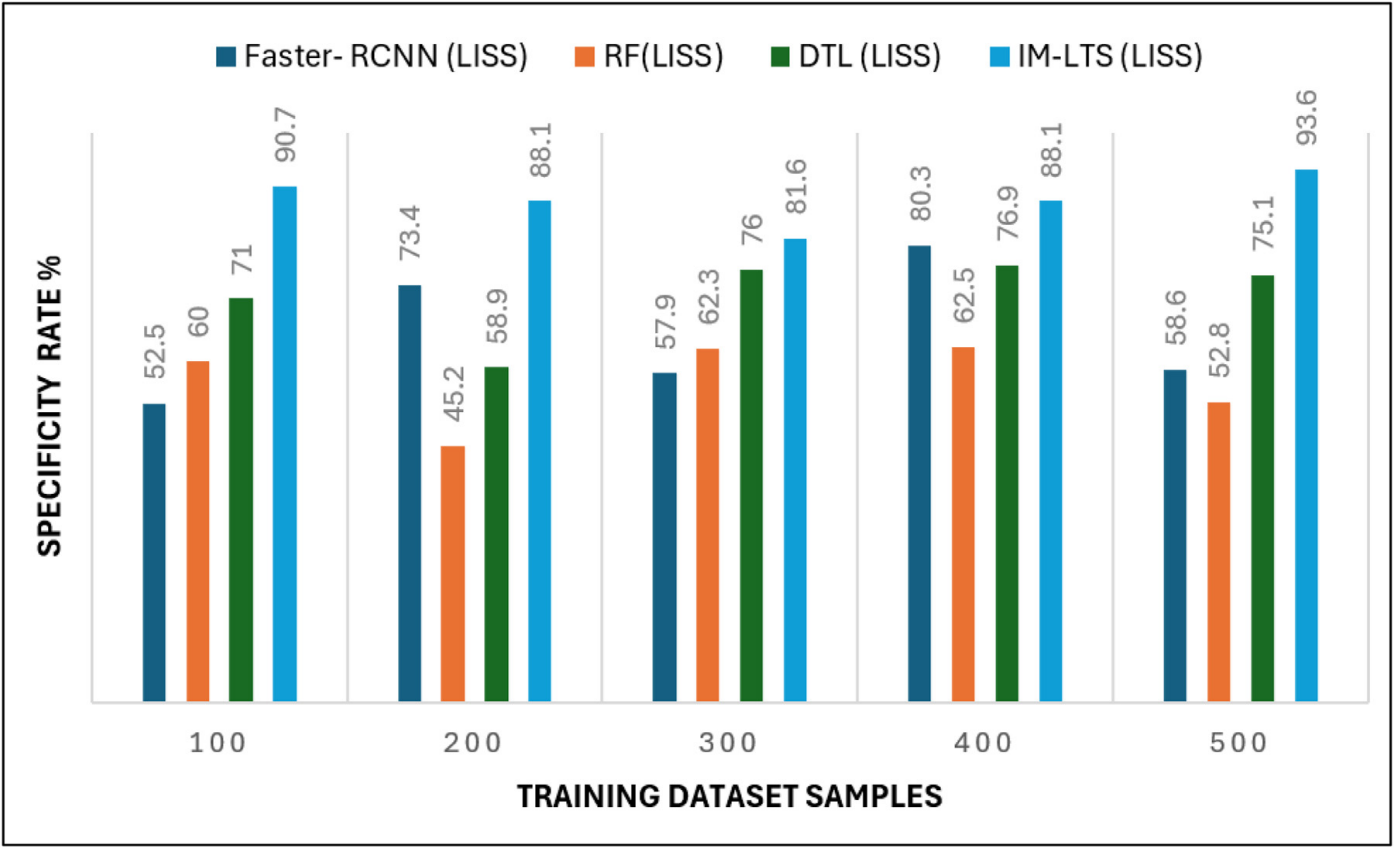
Specificity rate based comparisons and results for LISS.

The most crucial factors to consider when assessing a classification model for medical image diagnosis are accuracy and precision rates. Calculations are performed regarding that matter, and Fig. 8, Figs. 8a, Fig. 9 and Fig. 9a present the findings. Accurate diagnosis and categorization of lung cancer are necessary to enable radiologists to treat patients further. By using median and average filters effectively, a noiseless image is produced. Segmentation is then processed using a network model that is specifically designed to define the cancer tissue, and SVM-based classification is carried out to produce results with a higher classification accuracy rate. The accuracy rate for the proposed model is attained as {89.0, 93.7, 92.4, 92.0, 93.7} and {95.4, 96.3, 96.7, 95.4, 95.8}, for LDC and LISS dataset samples, respectively. The average error rate generated by the model is also regarded as a crucial component in determining the efficacy of any diagnostic model. Fig. 10 and Fig. 10a portray the error rate results produced by the proposed model compared with other works. The model produces {13.9, 10.5, 14.3, 8.4, 9.5} and {6.3, 13.8, 13.3,10.0, 7.9} error rates for LIDC and LISS, respectively. It can be evidenced from the figure that the proposed IM-LTS generates a minimal rate of error in classifying the tumor images with respect to both dataset images.

**Fig. 8:**
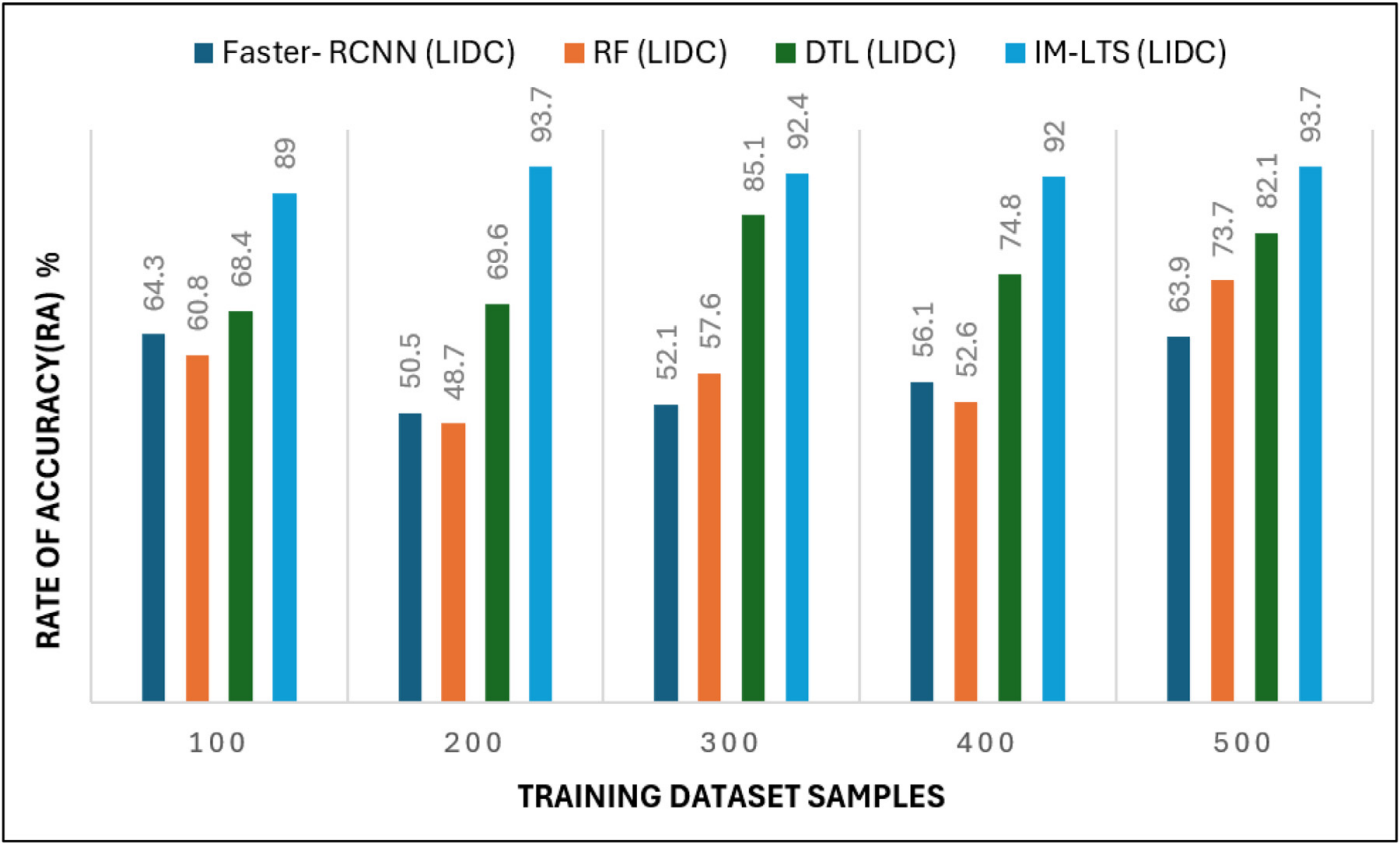
Accuracy rate in lung image classification for LIDC.

**Fig 8a:**
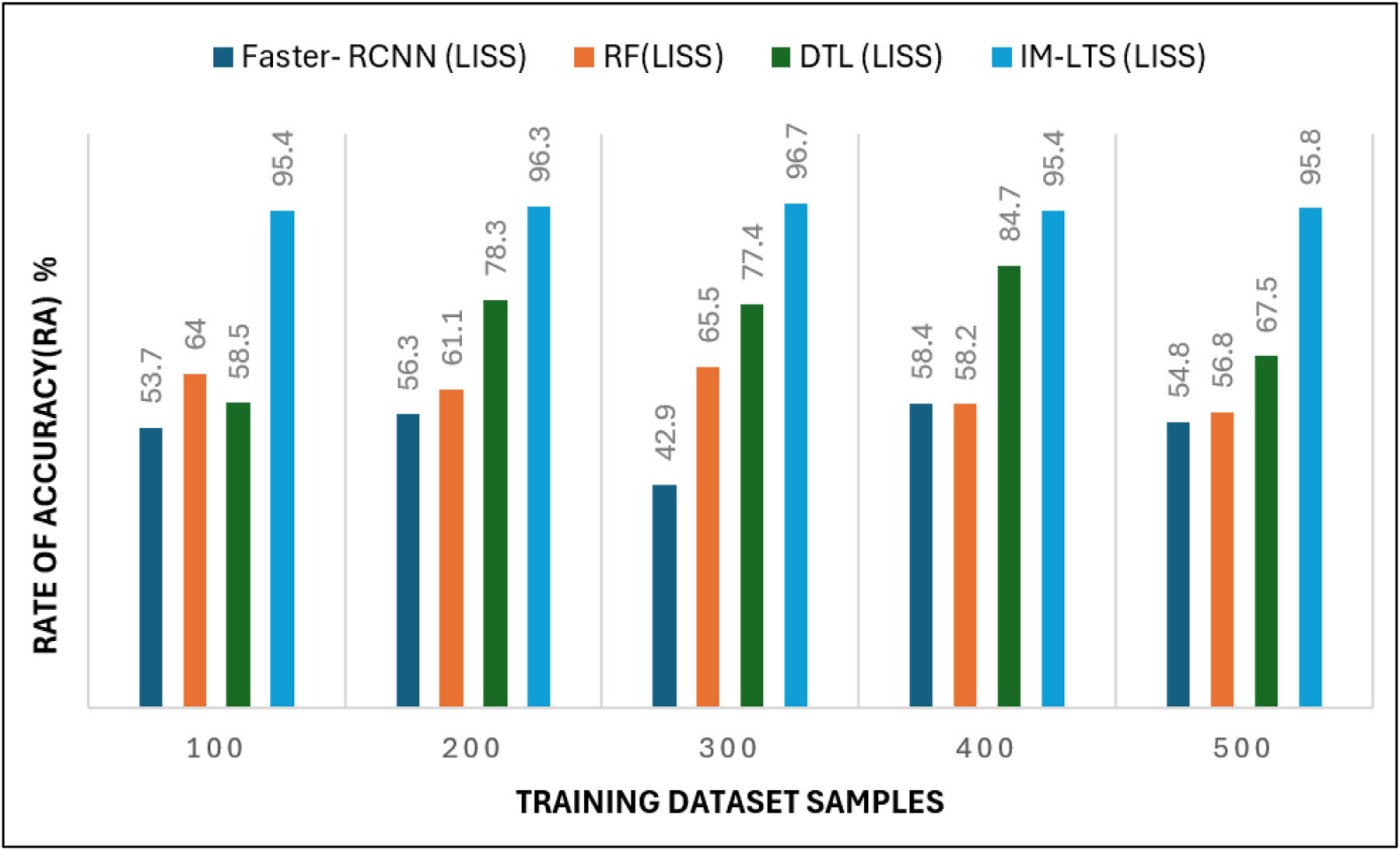
Accuracy Rate in Lung Image Classification for LISS.

**Fig. 9:**
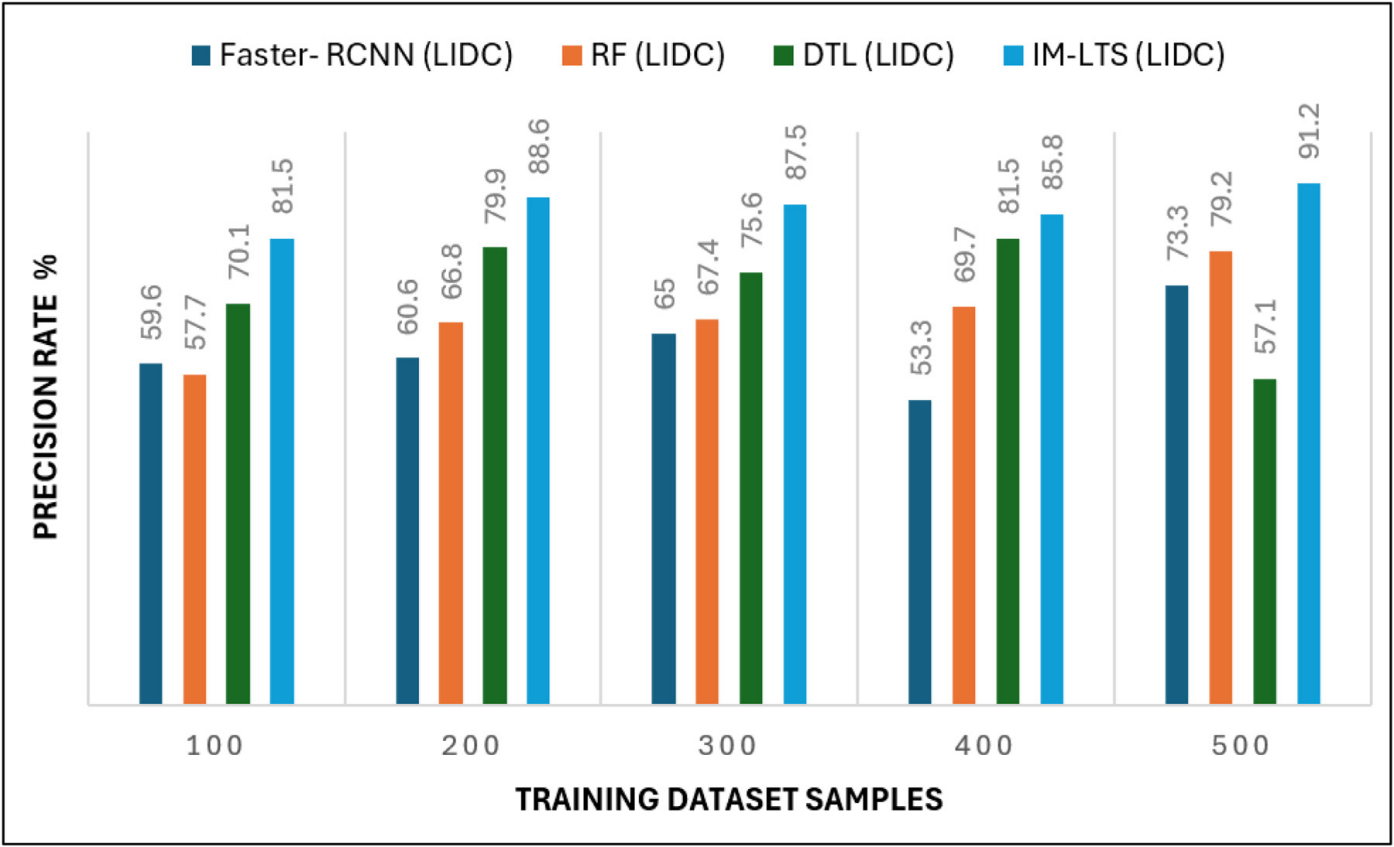
Precision rate analysis among models for LIDC.

**Fig 9a:**
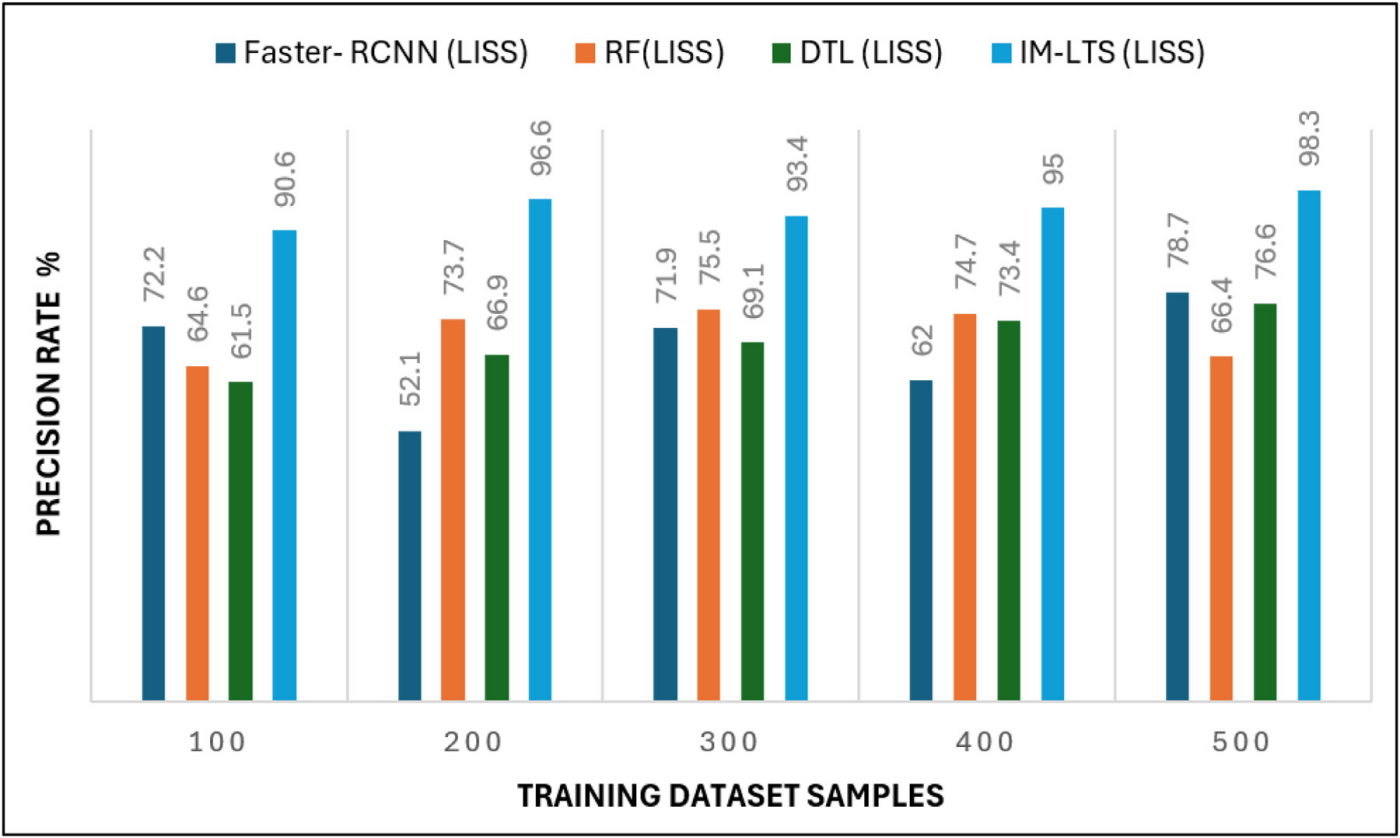
Precision rate analysis among models for LISS.

**Fig. 10:**
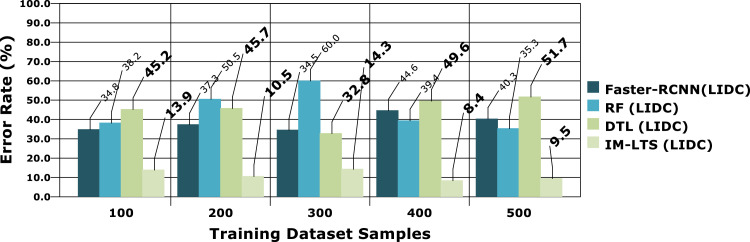
Error rate analysis among models for LIDC.

**Fig 10a:**
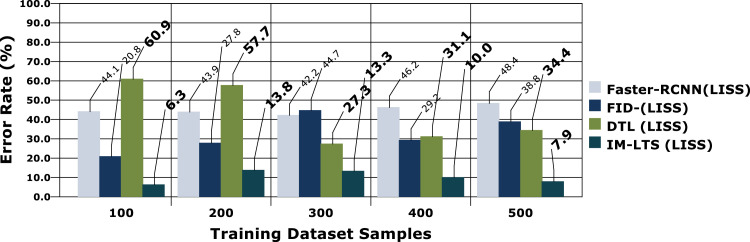
Error rate analysis among models for LISS.

This paper discussed how IoMT has made investigating and diagnosing diseases easier and more accurate. Here, the IM-LTS model is developed for diagnosing and evaluating lung tumor images, segmenting tumors, and classifying. The network model is designed using U-NET and MobileNetV2. Moreover, the inputs are acquired from IoMT sensors and CT scans, which are denoised for further feature extraction. The hyperparameters are extracted from the denoised images based on intensity, texture and shape-based features. The significant features from the images are given to the network model for segmentation. Further, SVM is used to classify the images into benign and malignant. The results for factors, sensitivity, specificity, precision, accuracy and error rates show that the proposed model provides better results than the compared works. The work can be enhanced in the future by employing transfer learning with different types of lung CT images. The current advanced technology can still be optimized for precise and timely disease diagnosis and quantitative evaluation.

## Limitations

The limitations of the proposed model include the dependence on variety of input images, which affect the generalizability. Paradoxically, the binary classification approach does not consider tumor grades and stages, and the computational model may be a challenge to implement in areas with limited resources. In future, improved pre-processed techniques need to be developed to handle noisy input.

## Ethics statements

This research does not involve human subjects.

## CRediT authorship contribution statement

**Jayapradha J:** Methodology, Software, Formal analysis, Investigation, Writing – original draft. **Su-Cheng Haw:** Validation, Resources, Conceptualization. **Naveen Palanichamy:** Conceptualization, Supervision. **Kok-Why Ng:** Validation, Writing – review & editing. **Senthil Kumar Thillaigovindhan:** Validation, Writing – review & editing.

## Declaration of competing interest

The authors declare that they have no known competing financial interests or personal relationships that could have appeared to influence the work reported in this paper.

## Data Availability

Data will be made available on request.
